# Comprehensive Annotation of the *Parastagonospora nodorum* Reference Genome Using Next-Generation Genomics, Transcriptomics and Proteogenomics

**DOI:** 10.1371/journal.pone.0147221

**Published:** 2016-02-03

**Authors:** Robert A. Syme, Kar-Chun Tan, James K. Hane, Kejal Dodhia, Thomas Stoll, Marcus Hastie, Eiko Furuki, Simon R. Ellwood, Angela H. Williams, Yew-Foon Tan, Alison C. Testa, Jeffrey J. Gorman, Richard P. Oliver

**Affiliations:** 1 Centre for Crop & Disease Management, Department of Environment and Agriculture, Curtin University, Bentley, WA, Australia; 2 Curtin Institute for Computation, Curtin University, Bentley, WA, Australia; 3 Protein Discovery Centre, QIMR Berghofer Medical Research Institute, Herston, Qld, Australia; 4 Telethon Kids Institute, Subiaco, WA, Australia; Youngstown State University, UNITED STATES

## Abstract

*Parastagonospora nodorum*, the causal agent of Septoria nodorum blotch (SNB), is an economically important pathogen of wheat (*Triticum* spp.), and a model for the study of necrotrophic pathology and genome evolution. The reference *P*. *nodorum* strain SN15 was the first Dothideomycete with a published genome sequence, and has been used as the basis for comparison within and between species. Here we present an updated reference genome assembly with corrections of SNP and indel errors in the underlying genome assembly from deep resequencing data as well as extensive manual annotation of gene models using transcriptomic and proteomic sources of evidence (https://github.com/robsyme/Parastagonospora_nodorum_SN15). The updated assembly and annotation includes 8,366 genes with modified protein sequence and 866 new genes. This study shows the benefits of using a wide variety of experimental methods allied to expert curation to generate a reliable set of gene models.

## Background

Although the cost of DNA sequencing has decreased to the point where it no longer represents a significant hindrance to obtaining an initial assembly, [1] an accurately annotated eukaryotic genome remains a significant challenge. Genome assembly can be hampered by errors arising from sequencing errors or the presence of repetitive regions, which can lead to truncated contigs and a fragmented assembly. Genes and other features are typically annotated using homology-based methods or are predicted *ab initio*. Experimental gene validation techniques are required to complement *in silico* methods to obtain high quality gene model annotations.

*Parastagonospora nodorum* [teleomorph: *Phaeosphaeria* (Hedjar.) syn. *Leptosphaeria nodorum* (Müll.*)*, syn. *Septoria nodorum* (Berk.), syn. *Stagonospora nodorum* (Berk.)] is a filamentous Ascomycete and member of the Dothideomycetes, a taxonomic class that includes several agriculturally-damaging phytopathogens [2–5]. *P*. *nodorum* causes the wheat disease Septoria nodorum blotch (SNB, syn. glume blotch) [6] and is responsible for substantial yield losses in many regions around the world. As part of the infection process, the fungus produces an arsenal of proteinaceous effectors that induce tissue necrosis and/or chlorosis on hosts expressing the corresponding susceptibility gene [7]. Analysis of the *P*. *nodorum* / wheat pathosystem has revealed the role of necrotrophic effectors SnToxA [8], SnTox1 [9] and SnTox3 [10] in conferring virulence. The presence of undiscovered effectors in *P*. *nodorum* is evident by observation of SNB QTLs in wheat cultivars when challenged with culture filtrate [11] from the reference strain devoid of known effector genes [12] or from other *P*. *nodorum* strains [3]. In addition to effectors, *P*. *nodorum* genes involved in primary metabolism, secondary metabolism, and signal transduction have been studied to elucidate their involvement in the *P*. *nodorum* pathogenic lifecycle. Characterised metabolic enzymes include malate synthase [13], δ-aminolevulinic acid synthase [14], pantoate-β-alanine ligase [15], trehalose 6-phosphate synthase [16] and components of mannitol metabolism [6, 17]. *P*. *nodorum* signal transduction and regulatory genes that have been studied in depth include the transcription factor *StuA* [18], a MAP kinase [19], calcium/calmodulin-dependent protein kinases [20], a putative short-chain dehydrogenases [21, 22] revealed to be necessary for the formation of the mycotoxin altenariol and sporulation [23] and those with a role in cyclic AMP signalling [24, 25].

The first published Dothideomycete whole genome assembly was of *P*. *nodorum* strain SN15. The original sequence was obtained in 2004 using 1 Kb, 4 Kb, and 40 Kb Sanger shotgun-sequenced paired reads assembled as 37.1 Mb of nuclear DNA in 107 scaffolds and the complete 49.8 Kb mitochondrial genome [26]. Initial gene-structure annotation relied heavily on automated methods, but was subsequently revised after analysis of proteogenomic [27], and microarray data [28] to give a total of 10,761 gene models with a mean exon count of 2.6, mean CDS length of 1,400 bp, mean intergenic distance of 1,685 bp, and a mean intron length of 91 bp. Repetitive sequence comprised 4.52% of the genome in 5 subtelomeric repeat classes, 1 ribosomal DNA repeat and 20 transposon or transposon-like clusters [29]. Repeat-induced point (RIP) mutations in repeat instances were subsequently *in-silico* reversed to allow classification of the repeat X26 as a RecQ helicase, R25 as a pseudogene, and repeats X3 and X8 as members of the same ancestral class [30].

The genomics resources available to *P*. *nodorum* researchers were expanded to include the genomes of two more strains—one isolated from the grass *Agropyron* that is unable to infect wheat, and a wheat-pathogenic isolate known to produce a different suite of effectors to the SN15 reference strain. In comparing the three strains, Syme et al. [31] added 1,621 ‘lower-confidence genes’ to the 10,761 genes from Bringans et al. [27] to minimise the possibility of missing potential effector loci, bringing the total number of putative genes used in that comparison to 12,382. Clustering of the predicted proteomes from the three strains revealed a core set of 10,464 conserved proteins and 2,421 putative proteins that were exclusive to strains able to infect wheat [31].

The initial SN15 assembly was found to contain a homolog of the *Pyrenophora tritici-repentis* necrotrophic effector ToxA, providing evidence of a horizontal gene transfer event from *P*. *nodorum* to *P*. *tritici-repentis* [8]. The *ToxA*-containing transfercon was initially estimated to be 11 Kb, however we propose that a larger region of at least 72 to 145 Kb was transferred [31, 32], corresponding to *P*. *nodorum* scaffolds 68, 55, 51, 46, 64, and 73.

The accuracy and completeness of a genome assembly can be improved by the addition of new sequencing data as error characteristics and shortcomings of one sequencing technique may be overcome by a complementary chemistry [33, 34]. The long read lengths available from Sanger sequencing have been useful to resolve repetitive regions and provide the large-scale structural assembly for SN15, however the depth and accuracy of additional Illumina short-reads could be used to correct remaining SNPs and small insertions or deletions [35]. Similarly, the quality of predicted gene models can be improved by the addition of transcriptomic and proteomic data sources by correction of errors in intron/exon boundaries and the revelation of new loci not previously known to be transcribed [27, 36].

Comparison of the chromosomes of filamentous ascomycetes have shown that related chromosomes tend to conserve gene content, but with shuffled gene order [37]. The resulting syntenic patterns are described as ‘mesosynteny’ and can be explained by frequent chromosomal inversions but infrequent translocations [37, 38]. Mesosyntenic patterns may also be utilized to resolve the order and orientation of scaffolds in a fragmented genome assembly and thereby identify groups of scaffolds that comprise a single chromosome [37]. The utility of this technique is most obvious when a reliable ‘finished’ genome can be used to improve a fragmented ‘draft’ assembly of a closely related species or strain.

This study offers greatly improved genome assembly and gene model annotation datasets for *P*. *nodorum* SN15 than the previous update described in Bringans et al. [27]. We report extensive correction of SNP and indel base-calling errors in the *P*. *nodorum* SN15 reference assembly, the closing of assembly gaps, extensive automated and manual gene annotation, and improvements to the functional characterisation of gene models. The new experimental data comprises RNA sequencing, DNA sequencing, and multiple sets of proteomic data which were used to inform comprehensive manual curation of gene models. Using these complementary approaches, we have generated a greatly improved genome assembly, and have re-predicted the gene and protein datasets including potential pathogenicity effector genes. These bioinformatic resources represent a substantial knowledge-base that will support future research in Dothideomycete genome biology.

## Methods

### Fungal culture

*P*. *nodorum* SN15 was maintained on V8-PDA medium. For the induction of extracellular proteins, 1 x 10^6^ *P*. *nodorum* SN15 spores were grown in Fries broth [39]. For genomic DNA, RNA and protein extraction experiments involving the intracellular and cell-wall/membrane sub-proteomes, 1 x 10^6^ *S*. *nodorum* SN15 spores were grown in minimal medium broth for 3 days [22]. The mycelium was harvested and freeze-dried prior to further manipulations.

### Genomic DNA extraction and Illumina sequencing

*P*. *nodorum* SN15 genomic DNA was extracted using a modified high-salt cetyltrimethylammonium bromide (CTAB) protocol [40]. Briefly, freeze-dried mycelia were ground to a fine powder using a chilled mortar and pestle. Genomic DNA was extracted using an extraction buffer that consisted of 100 mM Tris, 50 mM EDTA, 2M NaCl, 0.4% (v/v) β-mercaptoethanol, 2% (w/v) polyvinylpyrrolidone and 2% (w/v) CTAB. The genomic DNA was subjected to phenol/chloroform extraction, ethanol precipitation and washes. A paired-end library with an average insert size of 439 bp and read lengths of 100 bp was generated from SN15 genomic DNA and used for sequencing. Sequencing of the genomic DNA was carried by the Australian Genome Research Facility (Melbourne, Australia) using an Illumina HiSeq 2000 (Illumina, CA, USA).

### RNA extraction and Illumina sequencing

*P*. *nodorum* SN15 total RNA was extracted using the Trizol reagent (Invitrogen, CA, USA) and DNase-treated. PCR was used to check that the sample was free of genomic DNA [41]. RNA sequencing was carried out by Macrogen (Seoul, South Korea) using an Illumina HiSeq 2000.

Raw Illumina sequencing reads were inspected with FastQC [42]. Adapter sequence and low quality terminal sequences were removed with Cutadapt v1.0 [43]. Parameters and run details are available in S1 Text.

### Proteomic datasets

The extracellular proteome was extracted as described by Vincent et al. [44], using a modified tricholoacetic acid (TCA)/acetone protein precipitation procedure. Briefly, proteins from the extracellular culture filtrate were precipitated, collected by centrifugation and washed with 100% acetone. The protein pellet was subsequently air-dried at room temperature and suspended in 20 mM Tris pH 7. Residual TCA was progressively removed by dialysis of the suspension using D-Tube Dialyzer Maxi, MWCO 3.5 kDa (Novagen, Darmstadt, Germany) in several changes of 20 mM Tris pH 7 at 4°C for 48 hrs. Solubilised proteins were retained and stored at -80°C until further manipulation.

The intracellular proteome was extracted as previously described by the authors of this study [41]. Briefly, intracellular proteins from mechanically ground freeze-dried mycelia were solubilised in 20 mM Tris-Cl pH 7 and de-salted using a PD10 chromatography column (GE Healthcare, Little Chalfont, UK). Solubilised proteins were retained and stored at -80°C until required.

To facilitate cell wall/membrane (CWM) proteome extraction, freeze-dried fungal mycelia were ground with a mortar and pestle and washed three times with 20 mM Tris-Cl pH 7 to release and remove soluble intracellular proteins. The pellet was then washed three times with 0.1 M Na_2_CO_3_ to further remove soluble and peripherally-attached proteins. The pellet was then resuspended in 20 mM Tris-Cl pH 7 and subjected to 3 cycles of slow freeze and thaw to further break up the cellular material. Membrane-bound proteins were extracted using two methods: Extraction Procedure 1 (EP1)– 100 mg of membrane enriched pellet was extracted with 2% (w/v) SDS, 100 mM EDTA and 50 mM DTT in 100 mM Tris/HCl (pH7.8) by vortexing and boiling for 5 min followed by 5 min on ice (based on methods presented in Meijer et al., 2006 and Feiz et al., 2006); Extraction Procedure 2 (EP2)– 100 mg of membrane enriched pellet was extracted with 2% (w/v) SDS, 7 M urea, 2 M thiourea and 50 mM DTT in 125 mM triethylammonium bicarbonate (TEAB, pH 8.5) by vortexing and sonication for 15 min in an ice-cold sonication bath followed by resting for 30 min on ice. Vortexing and sonication steps were repeated. Subsequent sample processing for suspensions derived from ‘Extraction Procedures’ EP1 and EP2 were identical. Suspensions were centrifuged at 16,000 x g for 5 min (4°C) and the supernatants removed. Pellets were washed twice with either 100 mM Tris/HCl (pH 7.8) for EP1 or 100 mM TEAB (pH 8.5) for EP2. Respective supernatants were pooled, centrifuged at 20,000 x g for 15 min (4°C) and collected for further processing. Proteins were precipitated from supernatants by the addition 100% TCA to a final concentration of 20% (v/v) and incubated on ice for 30 min. Protein precipitates were harvested by centrifugation at 20,000 x g for 10 min (4°C). Pellets were washed twice with 90% (v/v) acetone and centrifuged each time as before. Protein pellets were briefly dried under a gentle stream of nitrogen and used immediately. The final pellets were re-suspended in 45 μL of EP2 extraction buffer (without DTT) and 5 μL of 1 M TEAB (pH 8.5) by repeated vortexing and incubating the tubes for 10 min in an ice cold sonication bath. Samples were centrifuged at 20,000 x g for 10 min (4°C) and supernatants collected for further processing. Protein concentration of all samples was determined using the 2D-Quant kit (GE Healthcare) according to the manufacturer’s ‘Standard procedure’ protocol. CWM proteins were digested without prior fractionation.

Intracellular and extracellular proteins were separated into 24 fractions using isoelectric point-based fractionation of proteins (Agilent 3100 Offgel fractionator) with liquid-phase recovery followed by digestion and LC-MS analysis of peptides. Offgel separations were performed using high resolution separation kits (pH range 3–10, 24 cm IPG gel strips; Agilent) and approx. 1 mg of protein per strip loading as described previously [45]. The pH of 100 μL aliquots of recovered Offgel fractions was adjusted by adding 10 μL of 1 M TEAB (pH 8.5). CWM sample aliquots of 80 μg protein were diluted with 10 μL of 1 M TEAB (pH 8.5) and the volume adjusted to 110 μL with EP2 extraction buffer (without DTT).

Proteins were reduced with 5 μL of 0.5 M tris(2-carboxyethyl)phosphine (TCEP, 22 mM final conc.) for 2.5 hours at 4°C and then alkylated with 16 μL of 1 M iodoacetamide (122 mM final conc.) in the dark at 22°C for 2 hours. Reducing agent TCEP was dissolved in 100 mM TEAB (pH 8.5) and neutralised with 10 M sodium hydroxide solution to pH 8. Sample proteins were co-precipitated with 1 μg of modified trypsin (Roche, sequencing grade) by adding 10 volumes of methanol as follows: One microliter of trypsin was added to the side of the Eppendorf tube and quickly flushed into the sample solution with 1.3 mL of 100% methanol at -20°C. Tubes were incubated overnight at -20°C. Protein precipitates were harvested by centrifugation at 20,000 x g for 15 minutes (4°C). Pellets were washed twice, once with 1 mL of 90% (v/v) methanol at -20°C and finally with 1 mL of 100% methanol at -20°C and centrifuged each time as before. Protein pellets were briefly dried under a gentle stream of nitrogen and continued immediately. The final pellets were re-suspended in 40 μL of 100 mM TEAB buffer (pH 8.5) containing 5% acetonitrile by repeated vortexing and incubating the tubes for 1 min in the sonication bath. Samples were incubated at 37°C for 2 hours followed by the addition of further 1 μg of modified trypsin and 6 hours (Offgel fractions) and 14 hours (CWM proteome) digestion at 37°C. Protein digests were stored at -80°C until analysis.

For LC-MS/MS analysis, trypsin-digested samples were centrifuged for 5 min at 20,000 x *g* and an aliquot of 5 μL (Offgel fractions) or 3 μL (CWM proteome) was diluted to a volume of 20 μL with 6.5% formic acid prior to injection. Tryptic peptides were separated on a Prominence nano HPLC system (Shimadzu, Kyoto, Japan) and data collected on a Hybrid LTQ Orbitrap mass spectrometer (Thermo Fisher Scientific, Bremen, Germany). Mobile phases for chromatographic peptide separation were as follows: Eluent A was milliQ water containing 0.1% formic acid and eluent B was 80% acetonitrile / 20% milliQ water (v/v) containing 0.1% formic acid.

Acidified Offgel fractions were loaded onto a reversed-phase trap column (Dionex Acclaim PepMap μ-Precolumn C18, 0.3 mm x 5 mm) at 30 μL/min in 100% eluent A for 3.5 minutes and subsequently separated on a reversed phase capillary column (Vydac Everest C18 5μm 300 Å, 150 μm x150 mm, Alltech) at 45°C and a flow rate of 1 μL/min. Separation was performed with gradients of 2–30% B over 60 and 90 minutes (depending on sample complexity), followed by a 95% B wash step, resulting in a total run time of 110 and 140 minutes, respectively. Data was either acquired on an LTQ Orbitrap Velos as outlined below or an LTQ Orbitrap XL as described in Hastie et al. [45] with the following modifications to data acquisition: target value of 1 x 10^3^ for ion trap MS/MS scans; dynamic exclusion set to 70 s; ion selection threshold 1000 counts.

Acidified CWM digests were loaded onto a reversed-phase trap column (ReproSil-Pur C18-AQ 3μm, 0.3 mm x 10 mm; Dr. Maisch, Ammerbuch-Entringen, Germany) and washed for 3.5 minutes at 30 μL/min using 100% eluent A. Peptide mixtures were subsequently back flushed onto a capillary column (150 μm x 150 mm) packed in-house with reversed-phase beads (ReproSil 100 C18 3μm; Dr. Maisch) and separated at a flow rate of 1 μL/min. Peptides were separated at 55°C using a sequence of linear gradients: to 5% B over 3.5 minutes; to 35% B over 166.5 minutes; to 45% B over 10 minutes; to 95% B over 10 minutes and then holding the column at 95% B for 10 minutes. Data was acquired on an LTQ Orbitrap Velos Pro as described below.

Column-separated peptides were electrosprayed into the LTQ Orbitrap Velos and LTQ Orbitrap Velos Pro through a Nanospray Flex Ion Source (Thermo Fisher Scientific) using 30 μm inner diameter uncoated silica emitter (New Objective). Spray voltage was 1.5 kV with no sheath, sweep or auxiliary gases used. The heated capillary temperature was set to 250°C and 285°C for the LTQ Orbitrap Velos and LTQ Orbitrap Velos Pro, respectively. An S-lens value of 50 to 55% was used.

The LTQ Orbitrap Velos (OT Velos) and LTQ Orbitrap Velos Pro (OT Velos Pro) were controlled using Xcalibur 2.2 software (Thermo Fisher Scientific) and operated in data-dependent acquisition mode to automatically switch between Orbitrap-full scan MS and ion trap- MS/MS acquisition. Full scan MS spectra (OT Velos: *m/z* 300–2000; OT Velos Pro: *m/z* 380–1700) were acquired in the Orbitrap mass analyser with a resolving power set to 30,000 (OT Velos) and 60,000 (OT Velos Pro) at 400 *m/z* after accumulation to a target value of 1 x 10^6^ in the linear ion trap. The top 20 (OT Velos) and 15 (OT Velos Pro) most intense ions with charge states ≥ +2 were sequentially isolated with a target value of 5,000 and fragmented using collision-induced dissociation (CID) in the linear ion trap. ‘Rapid’ scan mode was selected for the ion trap- MS/MS acquisition in the OT Velos Pro. Fragmentation conditions were set as follows: 35% normalized collision energy; activation q of 0.25; 10 ms activation time; ion selection threshold 1000 (OT Velos) and 5000 (OT Velos Pro) counts. Maximum ion injection times were 200 ms for survey full scans and 50 ms for MS/MS scans. Dynamic exclusion was set to 70 s and 90 s for OT Velos and OT Velos Pro runs, respectively. Lock mass of *m*/*z* 445.12 was applied with an abundance was set at 0%.

Mass spectra were then searched using the Tide search engine [46] implemented in the Crux toolkit [47] with specifications as follows: spectra mapped against: 6-frame translations of both the new and the old genome assemblies and the set of predicted protein sequences from both the new and the old annotations. The search parameters used were: variable modifications, oxidation (M); and deamidation (NQ); fixed modification, carbamidomethyl (C); peptide tolerance, 20 ppm; MS/MS tolerance: ±0.8 Da; Digestion enzyme: trypsin; maximum missed cleavages: 1. Peptide-spectrum matches were refined using Percolator [48], again as implemented in the Crux toolkit.

For 1D-LC MALDI MS/MS analysis of the SN15 extracellular proteome, SN15 trypsin-digested peptides were resuspended in 20 μl of 2% acetonitrile and 0.05% trifluoroacetic acid. Peptides were loaded onto a C18 PepMap100, 3 mm column (Dionex, CA, USA) through the Ultimate 3000 nano HPLC system (Dionex, CA, USA). Mass spectrometry analysis was carried out on a 4800 MALDI TOF/TOF Analyser as previously described [49]. These spectra were also searched using the Tide search engine [46] with specifications: variable modifications, oxidation (M); fixed modification, carbamidomethyl (C) and other parameters and post-processing as above.

Conflicts with existing annotations were identified where proteomic spectra searched against the six-frame translation of the genome mapped into intergenic regions, intronic annotations or coding regions in the wrong frame.

### Improvements to the SN15 Genome Assembly

SNP and indel errors in the *P*. *nodorum* SN15 assembly sequence [29] were corrected by MIRA (v3.4.1.1) [35], using its mapping algorithm to assemble Illumina gDNA reads onto the pre-existing scaffolds. The original Sanger-sequenced reads were also re-mapped to the corrected assembly using BWA v0.7.3a-r367 [27]. Groups of putative scaffold linkage groups were predicted by comparison to *Pyrenophora tritici-repentis* [32] using the synteny-based cumulative binomial test for mesosynteny described by Hane et al. [37].

In order to assess the outcomes of genome sequence and gene annotation corrections, various diagnostic tests were performed. Changes made to the corrected genome were calculated with the dnadiff tool distributed with MUMmer [50]. Improvements of WGS read mapping to the corrected assembly were calculated by alignment with BWA v0.7.5a-r405 using the default parameters and summary statistics calculated with Picard v1.9.4 [51]. Improvements of RNA read mapping to the corrected assembly were calculated by alignment with TopHat v2.0.12 [52] and summary statistics calculated from TopHat reports and with Picard.

### Improvements to the Genome Annotations

Errors in *P*. *nodorum* SN15 gene annotations were corrected using a combination of supporting data from RNA-seq and proteogenomic peptide alignments to the corrected assembly. RNA-seq reads were mapped to the corrected genome using TopHat v2.0.8 [52]. Manual correction of gene models and was performed using WebApollo [53]. JBrowse [54], through WebApollo was used to visualise the various ‘-omics’ data sources that informed the manual correction.

RNA sequencing reads were aligned to the genome, and the gene models identified by Bringans et al. [27] were checked to ensure they matched all introns supported by 5 or more RNA-seq reads. Introns were introduced or removed from the annotations to match the RNA-seq data. New genes were annotated where transcription levels exceeded 5X when a suitable open reading frame (ORF) could be found and/or the ORF included at least one conserved domain as predicted by InterProScan using the–pathways and–goterms arguments and default parameters otherwise. Gene annotations were split when the RNA-seq depth dropped to 0 and/or the concatenated protein’s BLAST hits showed two moieties of hit coverage. RNA-seq depth was also used to correct events where an ORF occurred inside the intron of another gene. These events were identified by large changes in read depth at a single locus. For each intronic insertion annotation, the translated region of the splice site skipping over the internal ORF was checked for consistency with blast results and with InterProScan-predicted domains spanning the splice site.

Exported and cleaned GFF3 and FASTA files were checked into git version control for distributed backup, sharing and review (https://github.com/robsyme/Parastagonospora_nodorum_SN15). Genome-wide support for gene annotations was summarised according to evidence type, requiring 80% coverage of coding sequence length and 5X coverage for RNA-seq support, peptides mapping within the coding region for proteogenomic support and four or more for microarray probes showing with expression levels at or above the cut-off determined by Ipcho et al. [15].

All gene annotations were manually reviewed and curated using the WebApollo platform, checking for consistency with RNA-seq, proteomics, microarray, BLAST hits against nr and conserved protein domain structures. Matches to conserved protein domains identified from translated gene models using InterProScan v5.8–49.0 [55] were compared between previously published and corrected datasets. Each protein set was submitted to dbCAN [56] for CAZyme enzyme family identification. GO functional annotations assigned by InterProScan were analysed for functional enrichment of the new protein set using the Fisher’s test implemented in the goatools package [57].

### Annotation and Comparison with Alternate Strains

*P*. *nodorum* strains SN4 and SN79 were re-annotated using Maker v2.31.8 [58]. Evidence supplied to Maker included the updated SN15 protein set and *ab-initio* predictions from the ab-initio mode of gene predictor CodingQuarry [59] using parameters generated from training on the updated SN15 annotations. The predicted protein set from the three *P*. *nodorum* strains were clustered using ProteinOrtho v5.11 [60] using the synteny option. Execution of parts of this analysis, including ProteinOrtho clustering, were aided by GNU parallel [61] and BioRuby scripts and gems [62, 63].

## Results

### Genome Assembly Sequence Correction

The genome of *P*. *nodorum* SN15 was re-sequenced using 100 bp paired-end Illumina libraries yielding 11.0 Gbp of raw sequence data equivalent to approximately 290x coverage. Short-reads were reassembled using the MIRA mapping algorithm to resolve or remove 37,501 Ns and correct 12,911 SNPs, 1,005 deletions, and 16,820 insertions (Table 1).

**Table 1:** Summary of corrections made to the *P*. *nodorum* SN15 genome assembly.

Description	Before	After	Change
**Number of nuclear scaffolds**	107	91	-16
**SNP changes**	0	12,911	12,911
**Single bp insertion corrected**^a^	0	16,820	16,820
**Single bp deletion corrected**^b^	0	1,005	1,005
**Unknown N sequences (bp)**	164,388	126,887	-37501
**WGS Reads mapping to genome (≥Q20)**	93,867,773	94,594,136	726,363
**WGS read mismatch rate (%)**^c^	0.5623	0.4851	-0.0772
**WGS indel rate (%)**^d^	0.0615	6.2e-03	-0.0553
**WGS reads aligned in pairs (%)**^e^	99.6402	99.6427	2.5e-3
**RNA Reads mapping to genome (≥Q20)**	5,872,361,103	10,842,396,864	4,970,035,761
**RNA indel rate (%)**^d^	0.0348	0.0043	-0.0305
**RNA reads aligned in pairs (%)**^e^	95.0119	96.1274	1.1155

^a^ deletion of erroneous sequence from the original assembly.

^b^ insertion of sequence missing from the original assembly.

^c^ rate of mismatched based relative to the reference sequence over all aligned regions.

^d^ number of short insertions/deletions observed in reads / total aligned bases.

^e^ percentage of reads with aligned mate pair.

The genome annotations as described by Bringans et al. [27] were supplied as input to the MIRA assembly so that gene coordinates and identifiers could be preserved despite the correction of insertions and deletions to the underlying assembly.

The corrected genome sequence allowed for an additional 726 Kb of DNA reads to be mapped. Similarly, an additional 4,970 Mb of RNA reads were mapped to the corrected assembly. The reads mapped with lower rates of mismatch (0.4851% for DNA), and insertions/deletions (0.0062% for DNA and 0.0043% for RNA). The number of reads mapping in concordant pairs increased to 99.6% for DNA and 96.1% for RNA (Table 1).

Proteomic mass-spectral peptide matches from extracellular, cell-wall/membrane-bound and intracellular protein fractions were pooled and matches isolated by more than 200 bp from another match were discarded as likely false-positives. Existing annotations were checked for reading-frame consistency with the remaining spectral matches and new proteins were annotated or existing annotations extended where spectral search results fell outside the coding regions.

Sanger-sequenced reads from previously generated 4 and 10 Kb plasmid and 40 Kb fosmid libraries [26] were aligned to the corrected assembly and paired-end information was used to reassess scaffold joining and orientation (Table 2). We identified read-supported scaffold pairings and orientation by filtering Sanger reads where each read in a pair mapped to a different scaffold, where each of the pairs mapped at only one position in the genome, and where each of the pairs mapped within 40 Kb of the scaffold ends. We excluded scaffold joins where multiple read pairs suggested conflicting pairs or orientation, leaving only unambiguous joins. This process linked 16 scaffolds. Scaffolds 76, 92, and 106 were identified by BLAST as misassembled high-identity matches (>95%) to the mitochondrial genome sequence and were excluded from the nuclear genome assembly.

**Table 2:** New scaffold joins improving the *P*. *nodorum* SN15 genome assembly. Joins were either predicted by mesosyntenic patterns or by terminal matches to long insert Sanger sequence reads. Orientations are indicated relative to that of scaffolds of the original assembly.

Center scaffold	Right scaffold	Orientation	Evidence
**scaffold_8**	scaffold_26	**→→**	Mesosynteny
**scaffold_29**	scaffold_48	**←←**	Mesosynteny
**scaffold_37**	scaffold_48	**→→**	Mesosynteny
**scaffold_51**	scaffold_55	**←←**	Mesosynteny
**scaffold_2**	scaffold_107	**←→**	Long-insert library
**scaffold_7**	scaffold_105	**→→**	Long-insert library
**scaffold_17**	scaffold_36	**→→**	Long-insert library
**scaffold_18**	scaffold_77	**←←**	Long-insert library
**scaffold_20**	scaffold_49	**→→**	Long-insert library
**scaffold_54**	scaffold_64	**←←**	Long-insert library
**scaffold_60**	scaffold_72	**←←**	Long-insert library
**scaffold_28**	scaffold_61	**←→**	Long-insert library
**scaffold_29**	scaffold_85	**→→**	Long-insert library
**scaffold_33**	scaffold_17	**←→**	Long-insert library

The repeat content of the new assembly was reassessed. Sub-telomeric repeats R22 and X48 [29] are modestly expanded in the corrected assembly, but repeat content remains largely unchanged (S1 Table).

### Gene Model Correction Summary

After genome corrections, there were 13,563 predicted nuclear genes (Table 3), of which 866 are new genes at new loci and 1,936 are confirmed genes that had been regarded as doubtful in earlier revisions. New loci have been numbered starting at 30,001.

**Table 3:** Summary of the characteristics of annotated *P*. *nodorum* SN15 genes and their protein products before and after manual re-annotation. Manually-annotated genes are longer, have more annotated transcripts, are more likely to accord with proteomic data, and are more likely to have conserved protein domains.

	Before	After
**Gene model count**	12,199	13,569
**Average exon count**	2.6	2.5
**Average CDS length (bp)**	1,271.4	1,368.7
**Intergenic distance mean (bp)**	600	1010
**Intergenic distance std dev (bp)**	1,616	2,057
**Intron length mean (bp)**	89.9	66.6
**Intron length std dev (bp)**	84.3	52.4
**Proteins with Pfam domains**	11,464	13,248
**Proteins with Gene3D domains**	11,287	13,184
**Proteins with SignalP predictions**	1,122	1,476
**Models with peptide support**	2,665	4,352
**Models with peptide conflict**	150	0
**Genes with alternative transcripts**	0	299

**Fig 1:**
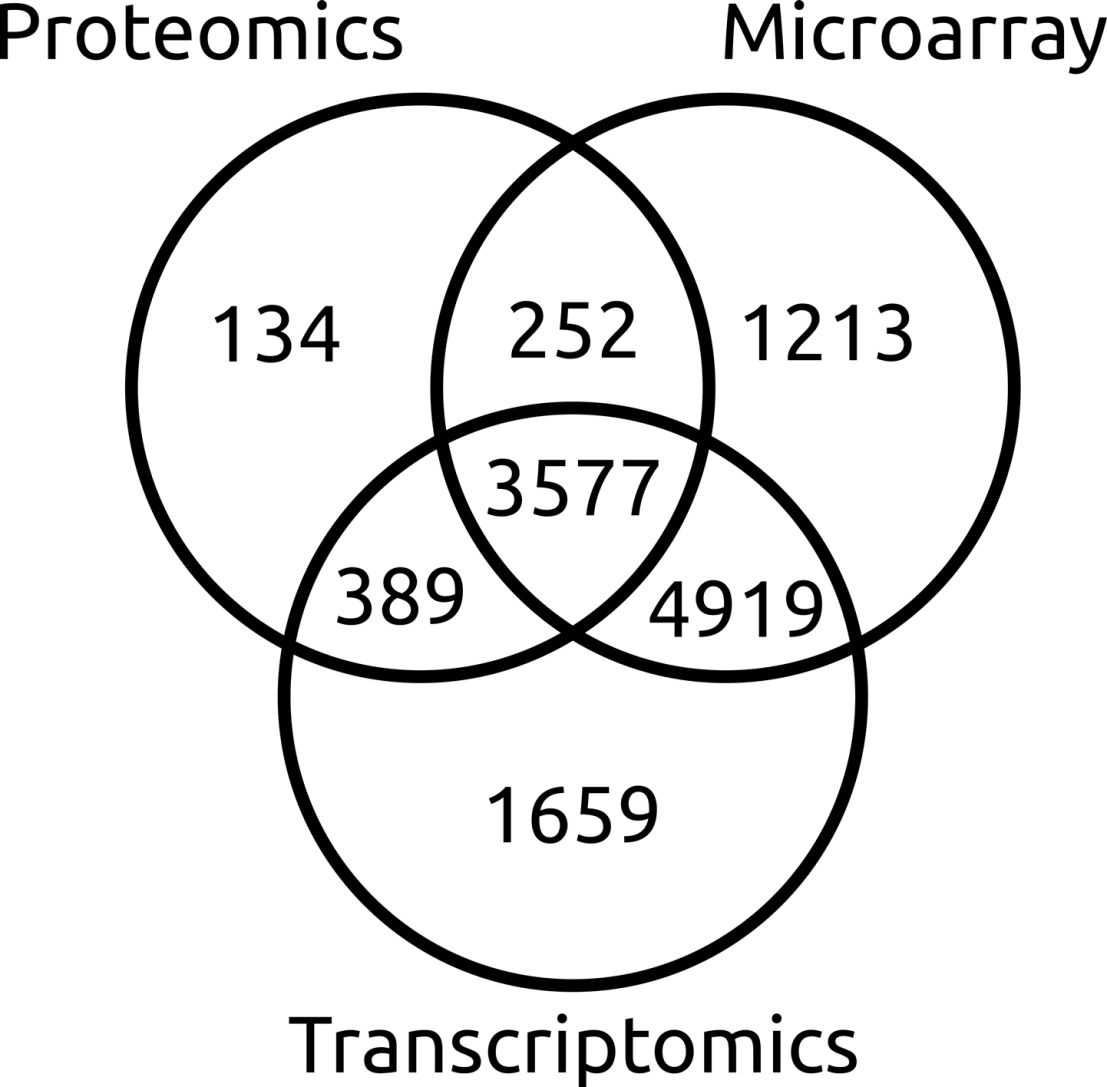
Sources of evidence used to re-annotate *P*. *nodorum* SN15 genes. This data supported 12,143 annotations with at least one source of experimental support. Additional annotations were also supported by non-experimental sources including the presence of conserved domains or homology to genes of other species.

In total, 12,143 (89%) genes in the current list possess some form of experimental supporting evidence (Fig 1). Microarray probe intensity supported the transcription of 9,961 loci. RNA-seq supported the exon structure of 10,544 gene models, including 299 loci with at least one alternatively spliced transcript, encoding a total of 13,949 proteins. 8,366 existing genes have had their protein sequence modified, 1,936 previously deprecated loci have been reinstated, and 866 new genes were introduced when the previous genome annotation had incorrectly split genes (55 occurrences, S2A Table), joined genes (356 occurrences, S2B Table) or where there was no previous annotation (455 occurrences) (Table 3, S3 Table). Four intronic endonuclease [64, 65] insertion events were annotated where an open reading frame occurred within another gene (*SNOG_30297*, *SNOG_30841*, *SNOG_14322*, and *SNOG_16073*). BLAST analysis of the inserted endonuclease protein sequences to the NCBI non-redundant protein database returned only hits to fragments of loci annotated as the host gene.

A core set of 11,849 protein clusters present in all three *P*. *nodorum* strains were identified by combining orthologous proteins using ProteinOrtho. Inputs were the improved set of SN15 annotations (13,949 proteins) and the proteomes of SN4 and SN79 re-annotated based on the new reference gene models (13,899 proteins and 13,746 proteins respectively).

### Functional Annotation Improvements

**Fig 2:**
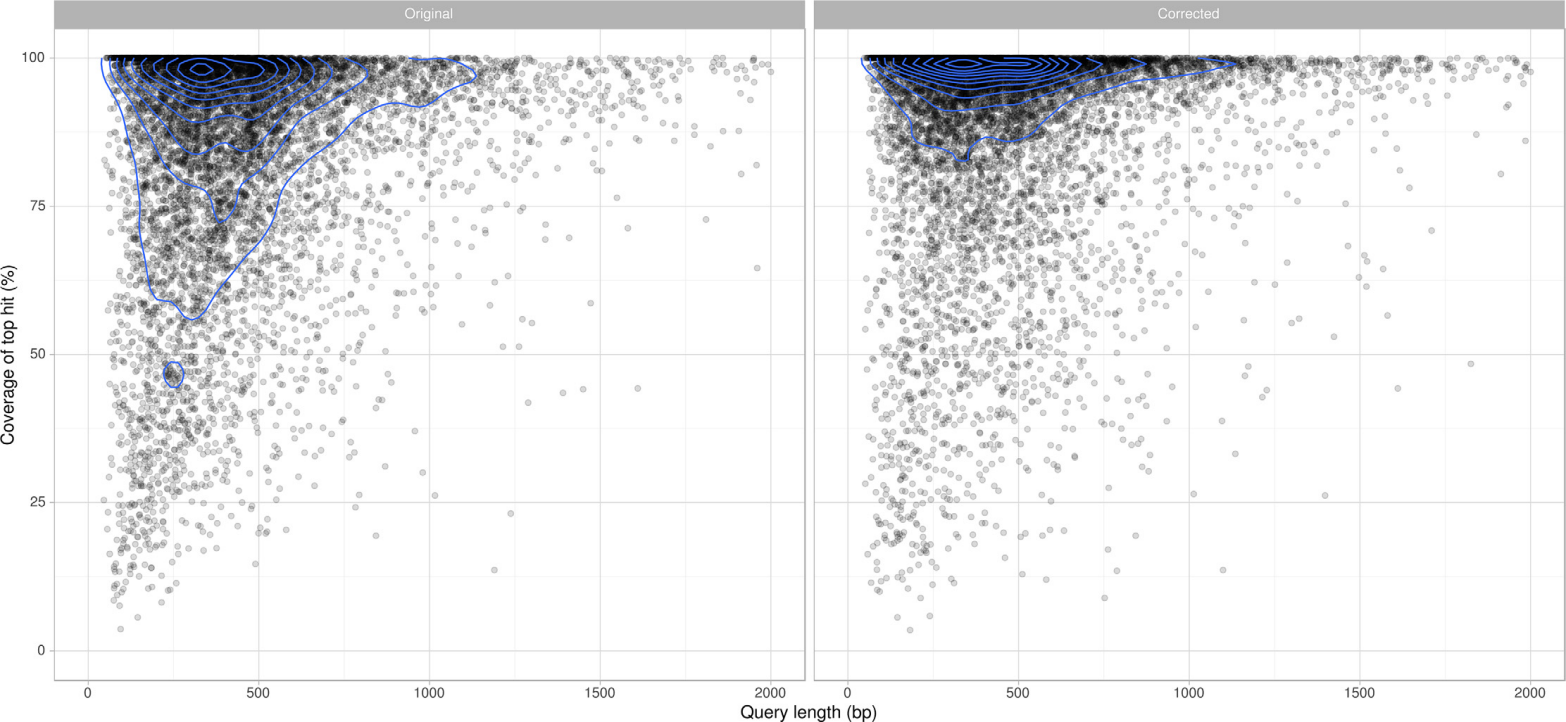
Coverage of the top BLASTP hit for re-annotated *P*. *nodorum* SN15 predicted proteins. The manually curated set (left) agrees more closely with sequences in the NCBI Protein NR database than the original set of annotations (right). Contour lines (blue) indicate ‘kernel density’, depicting the relative number of proteins within a localised region of the plot.

Comparison of each predicted protein to their top BLAST hit not belonging to the *Parastagonospora* genus reveals the new annotation set to be more concordant with annotations in other species (Fig 2). In particular, we observed a dramatic shift from shorter annotations to longer annotations that represent a higher proportion of the length of their best-matching homolog. Manual correction has eliminated occurrences of conflict between the predicted protein set and the mapped location of proteomic spectra (Table 3).

Compared to the gene annotations from Bringans et al. [27], the new set includes 1,784 more proteins with predicted Pfam domains [66], 1,897 more with Gene3D domains [67], and 354 more with SignalP-predicted signal peptides [68] (Table 3). CAZyme classifications show an increase in the number of proteins belonging to the carbohydrate-binding module (46), carbohydrate esterase (32), glycoside hydrolase (16), and glycosyl transferase (9) families after re-annotation (Table 4).

**Table 4:** Summary of carbohydrate-active enzyme (CAZyme) family numbers in *P*. *nodorum* SN15 before and after manual re-annotation.

CAZyme Family		Original match count	Corrected match count
**Auxiliary Activity**	AA	122	139
**Carbohydrate-Binding Module Family**	CBM	64	110
**Carbohydrate Esterase Family**	CE	142	174
**Dockerin**	-	1	1
**Glycoside Hydrolase Family**	GH	264	280
**Glycosyl Transferase Family**	GT	96	105
**Polysaccharide Lyase**	PL	10	10

### Genes and Domains of Interest

**Fig 3:**
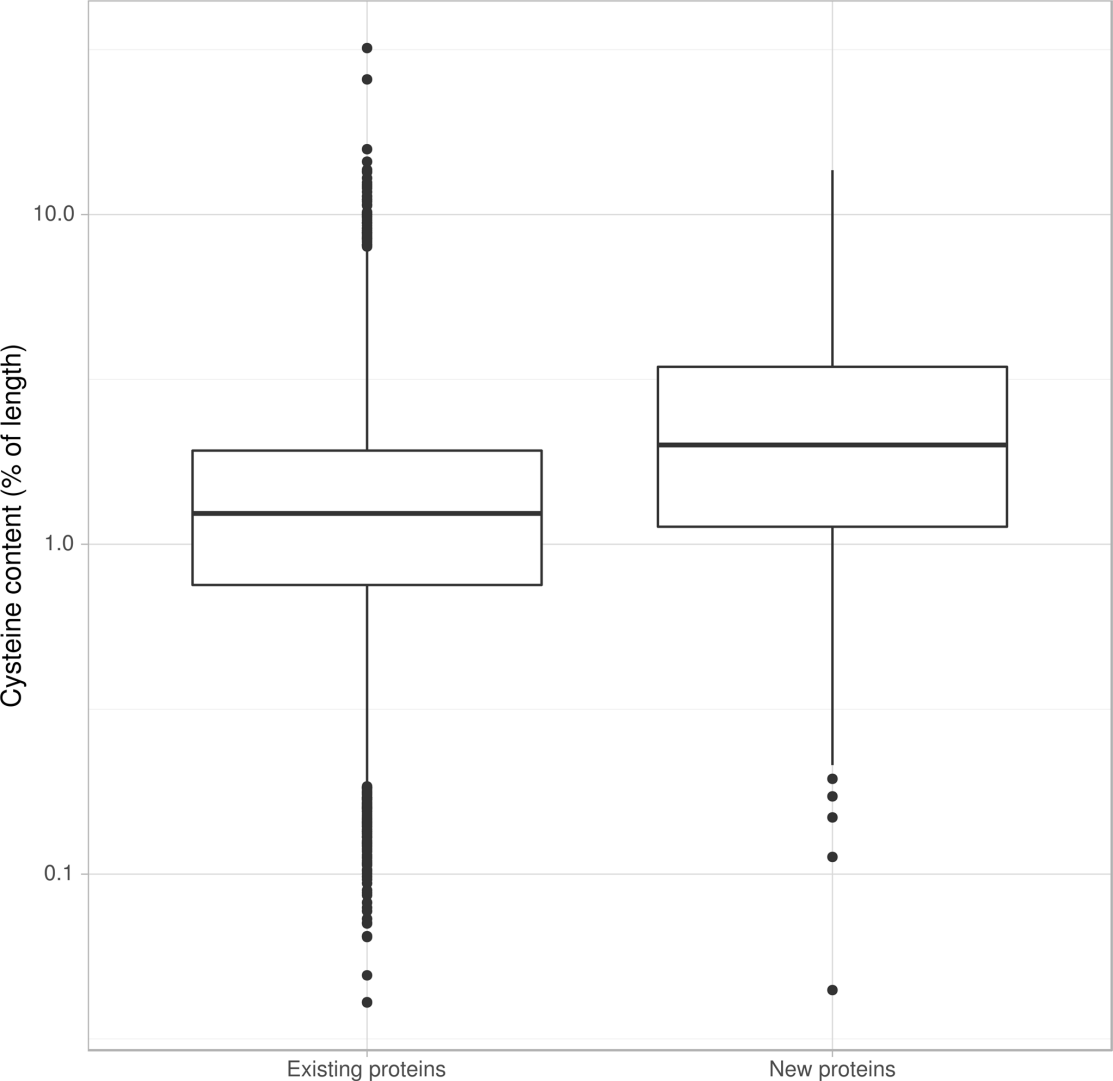
Proportion of cysteines in *P*. *nodorum* SN15 predicted proteins before and after gene re-annotation. New proteins are more likely to be cysteine-rich. Of the 54 cysteine-rich proteins in the new annotation set (> 9% Cys by length), 16 are the products of newly annotated loci.

Known *P*. *nodorum* effectors ToxA, Tox1, and Tox3 are not homologous but do share common characteristics. They are small (13 kDa, 10 kDa and 17 kDa respectively), contain signal peptides to target the protein to the secretory system and contain cysteine residues which may form disulphide bridges that help maintain protein stability once secreted. Their genes are positioned close to repeats. It has been suggested that effector proximity to repeats may expose them to an elevated level of mutation due to leakage of the RIP process outside truly repetitive sequence [69]. The known *P*. *nodorum* effectors are absent from the SN79 strain, and are highly expressed early in infection [15]. Among the 866 proteins annotated at new loci are proteins enriched in the properties of known necrotrophic effectors. This includes elevated cysteine content that may facilitate disulphide bridge formation for extracellular structural stability [70]. The newly annotated proteins have products with higher average cysteine content than the unchanged or modified proteins (Fig 3). Of the 54 proteins in the corrected set with more than 9% cysteine content, 16 are from genes at previously unannotated loci, and 51 have no BLAST match in to the NCBI Protein database (Table 4). The corrected set revealed 187 extra proteins with BLAST hits to entries in the PHIbase pathogen-host interaction database [71] that are experimentally shown to influence pathogenicity. Included among the cysteine-rich genes at new loci is a putative degraded copy of *P*. *nodorum* effector gene *Tox1* (Table 5, S4 Table). Effectors and other components of pathogenicity are likely to be members of the set of 2,169 protein clusters present in at least one wheat pathogen but absent from the avirulent SN79 strain.

**Table 5:** Summary of cysteine-rich protein-products of previously unannotated genes in *P*. *nodorum* SN15. Novel cysteine-rich annotations have few BLAST hits and include potential effector candidate genes e.g. *SNOG_30451*- a degraded and truncated homolog of *Tox1*.

Gene name	Protein length	Cysteine count	Cysteine percentage	Blast hits
***SNOR_30077***	66	9	13.6	No
***SNOR_30525***	74	10	13.5	No
***SNOR_30316***	94	11	11.7	No
***SNOR_30335***	70	8	11.4	No
***SNOR_30888***	53	6	11.3	No
***SNOR_30837***	56	6	10.7	No
***SNOR_30741***	355	37	10.4	Carbohydrate-binding
***SNOR_30253***	58	6	10.3	No
***SNOR_30352***	79	8	10.1	No
***SNOR_30019***	60	6	10	No
***SNOR_30451***	62	6	9.7	Fungal hypothetical genes
***SNOR_30925***	104	10	9.6	No
***SNOR_30828***	84	8	9.5	No
***SNOR_30466***	84	8	9.5	Tox1
***SNOR_30530***	76	7	9.2	No
***SNOR_30989***	55	5	9.1	No

All but one of the polyketide synthase (PKS) genes have had their gene structure modified (Table 6, S1 Fig). The modified protein models were used by Chooi, Muria-Gonzalez [72] to identify 24 PKS genes with one type III PKS, one hybrid non-ribosomal peptide synthetase/PKS, one partially reducing PKS, 7 non-reducing PKSs, and 14 highly reducing PKSs. Two extra proteins with putative pathogenicity domains HCE2 [Pfam: PF14856] and Ricin-type beta-trefoil lectin [Pfam: PF00652] are uncovered in the new protein set (Table 7, S4 Table), the latter of which has also been identified as a potentially important pathogenicity factor in another fungal wheat pathogen *Rhizoctonia solani* AG8-1 [73]. Overall, Pfam domains with an increased representation in the new protein set include DNA-binding domains (117), transcription factors (51) and chitin-binding sequence (21).

**Table 6:** Polyketide synthase genes of *P*. *nodorum* SN15.

Gene name	PKS Type
***SNOG_09622***	Type III PKS
***SNOR_00308***	Hybrid Nonribosomal peptide synthetase/PKS
***SNOR_00477***	Partially reducing-PKS
***SNOR_02561***	Highly reducing-PKS
***SNOR_04868***	Highly reducing -PKS
***SNOR_05791***	Highly reducing -PKS
***SNOR_06676***	Highly reducing -PKS
***SNOR_07866***	Highly reducing -PKS
***SNOR_09623***	Highly reducing -PKS
***SNOR_11066***	Highly reducing -PKS
***SNOR_11076***	Highly reducing -PKS
***SNOR_11272***	Highly reducing -PKS
***SNOR_12897***	Highly reducing -PKS
***SNOR_13032***	Highly reducing -PKS
***SNOR_14927***	Highly reducing -PKS
***SNOR_15965***	Highly reducing -PKS
***SNOG_09490***	Highly reducing -PKS
***SNOR_06682***	Non-reducing -PKS
***SNOR_07020***	Non-reducing -PKS
***SNOR_11981***	Non-reducing -PKS
***SNOR_15829***	Non-reducing -PKS
***SNOR_08274***	Non-reducing -PKS
***SNOG_08614***	Non-reducing -PKS
***SNOG_09932***	Non-reducing -PKS

**Table 7:** Summary of changes to protein-products with functional annotations of high relevance to plant pathogenicity in *P*. *nodorum* SN15, before and after re-annotation.

Pfam ID	Domain name	Before	After
**PF14856**	Hce2	**1**	**2**
**PF00652**	Ricin_B_lectin	**0**	**1**
**PF00188**	CAP	4	4
**PF10167**	NEP	0	0
**PF01476**	LysM	2	2
**PF05630**	NPP1	2	2
**PF11584**	Toxin_ToxA	1	1

## Discussion

The completeness and accuracy of an organism’s reference genome sequence and its gene annotations directly influence the validity of computational and reverse genetics-based downstream functional studies. This is especially relevant in plant pathology, for which considerable research efforts are invested into predicting and functionally characterising putative effector genes from genomic datasets. Identification of effectors and subsequent effector-assisted breeding programs have been an important contribution to crop protection against pathogens [74]. Screening of potential lines with a purified effector negates or diminishes the need for more costly and time-consuming infection assays and field trials. Analysis based on protein sequence such as effector prediction or functional annotation rely on accurate gene models, and by extension, assembly sequence.

For example, insertion and deletion errors in the underlying assembly sequence can force automated gene calling software to introduce erroneous intron features in order to extend an open-reading frame. This can lead to an inflated exon count and interrupt BLAST and/or protein domain matches, which can impair assignment of biologically relevant functional terms to genes [73].

**Fig 4:**
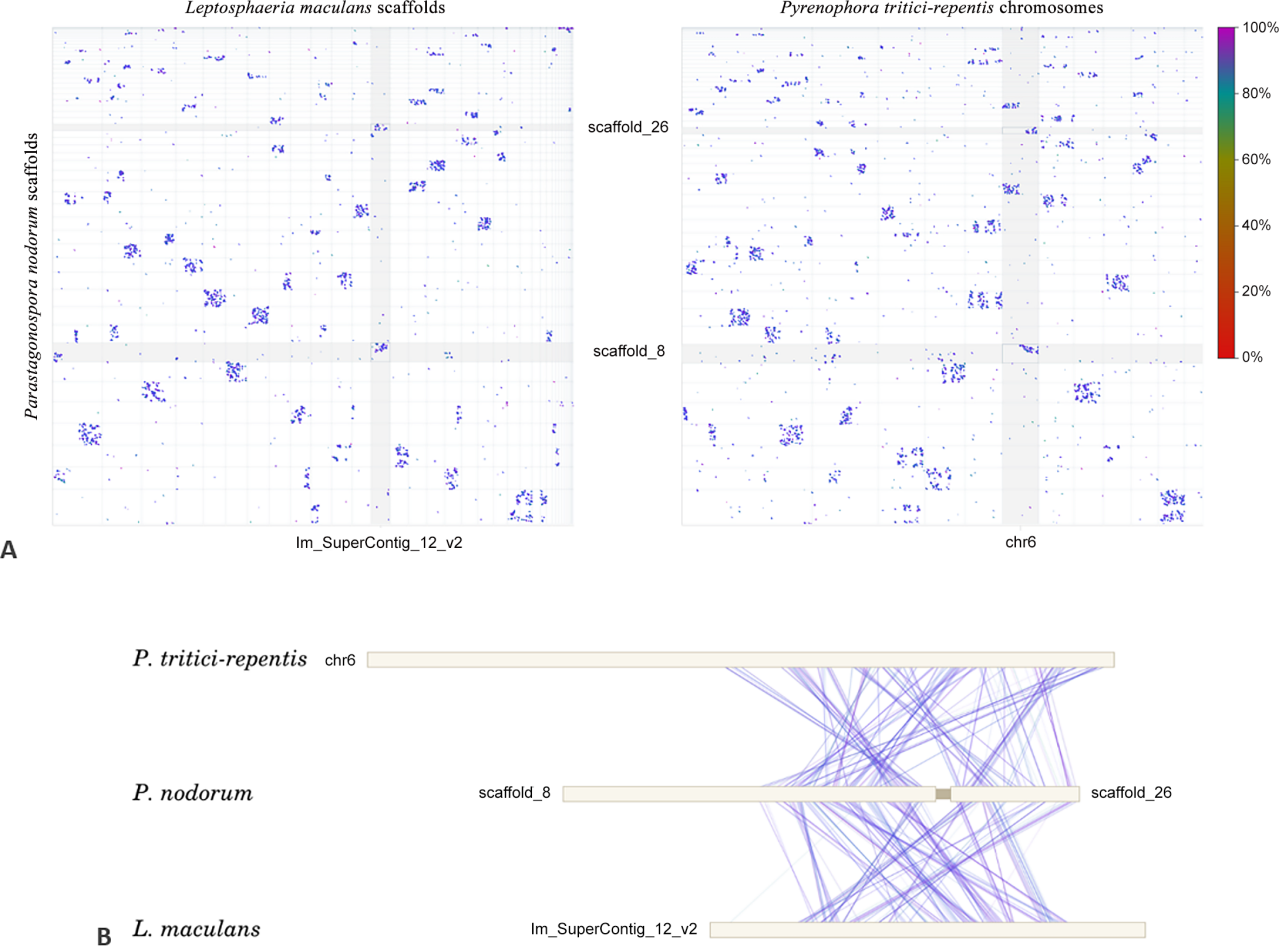
A whole-genome dotplot of nucmer matches between scaffolds of *P*. *nodorum* and of *P*. *tritici-repentis*. The ‘dots-in-boxes’ pattern is indicative of mesosyntenic relationships between chromosomes. *P*. *nodorum* scaffolds 8 and 26 are ‘mesosyntenic’ versus *P*. *tritici-repentis* scaffold 4, as indicated by black boxes.

A number of corrections have been made to the *P*. *nodorum* SN15 genome assembly, reducing the number of nuclear scaffolds from 107 to 91. SNP and indel removal facilitated by the addition of the Illumina data allowed a re-evaluation of the long-range paired end Sanger read data which, in turn, permitted the confident joining of 8 pairs of scaffolds. Eight scaffolds were joined that exhibited mesosyntenic relationships (Table 2). Scaffolds 8 and 26, for example both show mesosyntenic similarity to scaffold 4 on *P*. *tritici-repentis* (Fig 4). Joining scaffolds adds to our knowledge of the genomic context of particular regions of the genome, including the ‘transfercon’ harbouring the *ToxA* gene [8, 31, 32]. Confirmation of the scaffold 55/51 join predicted by mesosyntenic pairings and by homology to the *ToxA* region in *P*. *tritici-repentis* [31] lends support to the theory of an expanded 72 Kb transfercon and subsequent repeat invasion in *P*. *nodorum*. Pulsed field gel electrophoresis has been previously used to resolve between 14 and 19 chromosomes from different *P*. *nodorum* isolates [75]. Hence, a substantial number of gaps still remained unresolved in the current assembly.

In addition to improvements in existing genes, manual annotation has also uncovered genes at new loci. Many of these new genes are small and cysteine-rich (Table 5) with few BLAST hits to the NCBI protein database–hallmark characteristics of proteins involved in pathogenicity [31] and are effector candidates. Further evidence that these are relevant effectors could be obtained by determining whether they are expressed *in planta*.

The errors in the *P*. *nodorum* SN15 assembly sequence and its genome annotations are not unusual for a genome project of its age, assembly strategy and sequencing history. Similar fungal genome projects lacking ‘multi-omics’-based evidence may therefore harbour undiscovered annotation and sequencing errors, adversely affecting the accuracy of their genome analysis and the accuracy of comparative genomics studies in which they have been used.

We present an integrated analysis of multiple genomic, transcriptomic and proteomic datasets and their application to the improvement of the genome assembly and gene annotations of the fungal pathogen *P*. *nodorum* SN15. Experimental approaches undertaken in this study can readily be applied to other biological systems to refine gene models and assist in the assembly of uncompleted genomes. We anticipate that others establishing fungal genome projects would similarly benefit from the techniques described in this study.

## Supporting Information

S1 Fig Details of polyketide synthase genes before and after correction. (DOCX)

S1 Table Re-annotated repetitive content in updated genome assembly of *P*. *nodorum* SN15. (DOCX)

S2 Table Details of modified gene annotations. A) Genes that are a product of merging two or more annotations. B) Genes that are a product of splitting one annotation into two or more genes.
(DOCX)

S3 Table Details of new gene annotations. (DOCX)

S4 Table Details of cysteine-rich protein properties, matches to PHIbase and changes in functional annotations. (DOCX)

S1 Text Supplementary Methods: Illumina read QC and trimming parameters. (DOCX)
