# Supplementary material for: Comprehensive Annotation of the Parastagonospora nodorum Reference Genome Using Next-Generation Genomics, Transcriptomics and Proteogenomics
Source: PLoS One. 2016 Feb 3;11(2):e0147221. doi: 10.1371/journal.pone.0147221 (PMC4739733; doi:10.1371/journal.pone.0147221)
Supplement: S1 Fig — (DOCX) [file pone.0147221.s001.docx]

## S1 Figure | PKS gene models before and after correction

PKS gene models before and after correction. Coding sequence is shown in yellow. Disagreements between the underlying nucleotide sequences are shown as black regions in the grey bars. Indel errors in the underlying sequence force the gene prediction algorithms to introduce false introns.

*
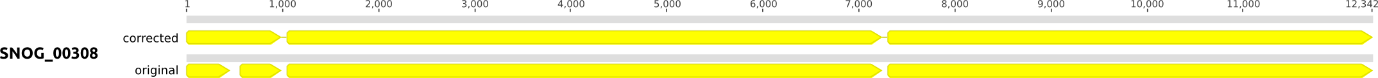
*

*
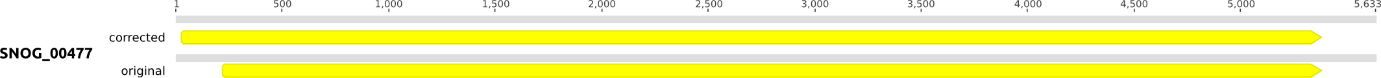
*

*
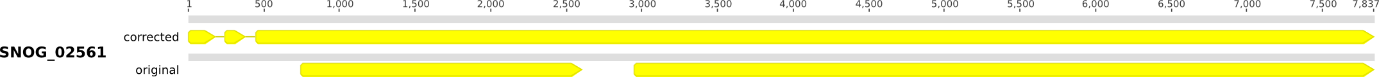
*

*
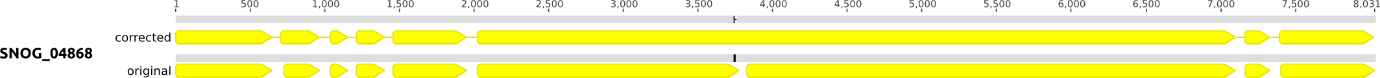
*

*
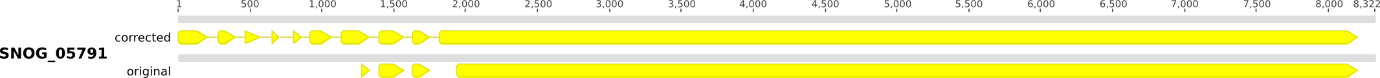
*

*
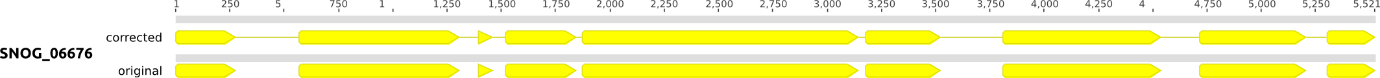
*

*
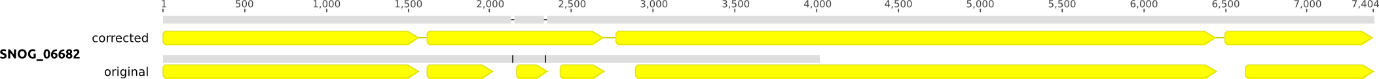
*

*
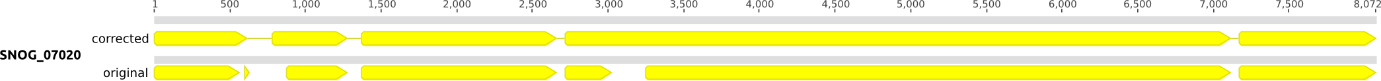
*

*
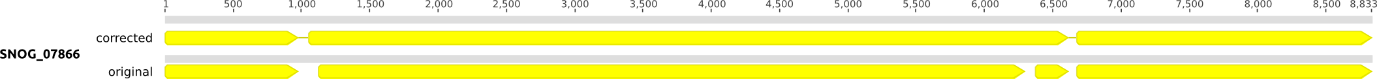
*

*
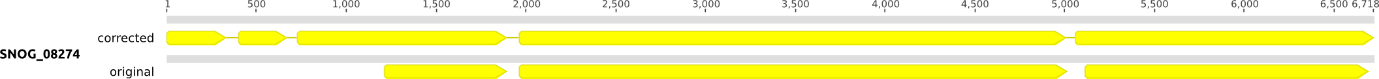
*

*
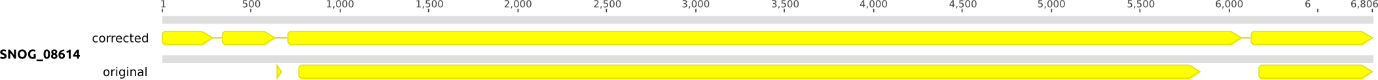
*

*
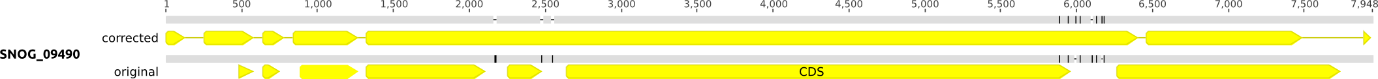
*

*
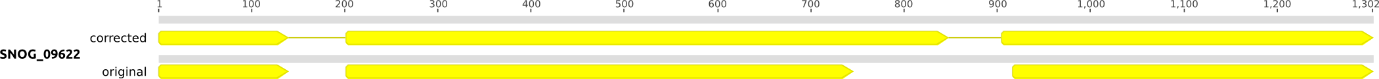
*

*
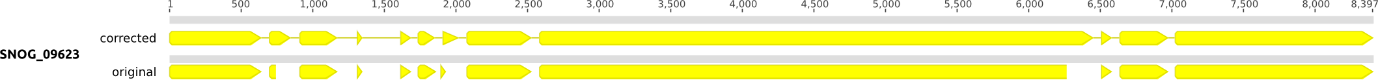
*

*
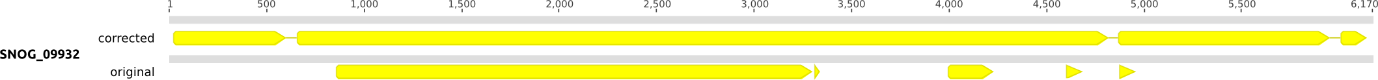
*

*
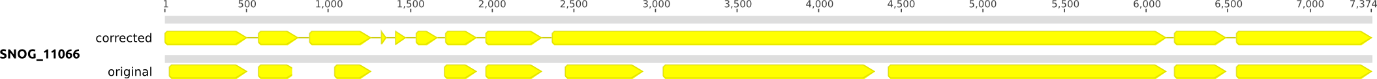
*

*
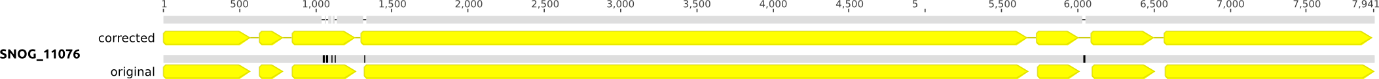
*

*
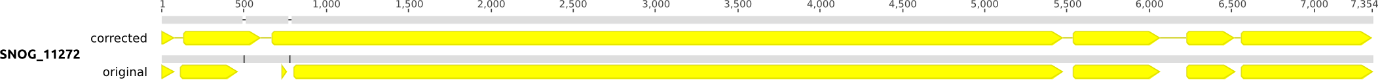
*

*
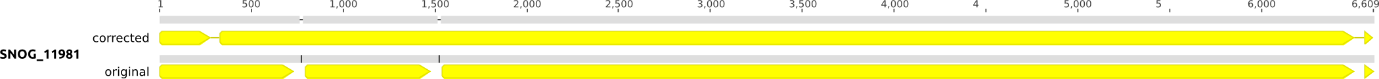
*

*
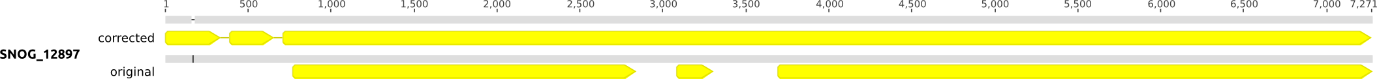
*

*
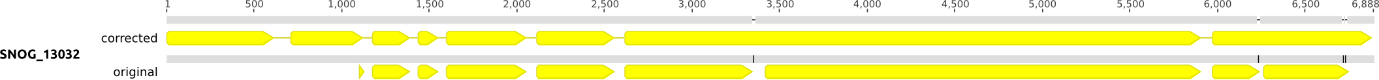
*

*
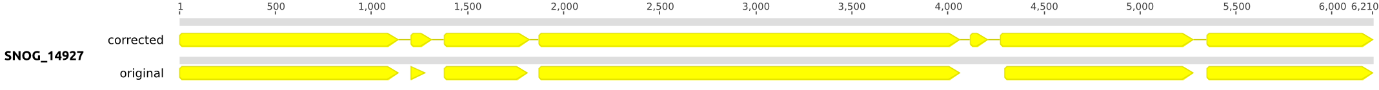
*

*
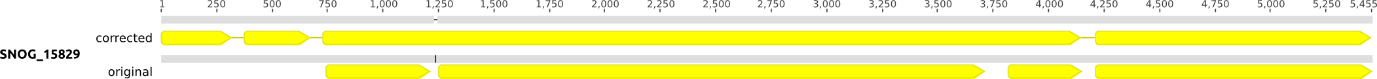
*

*
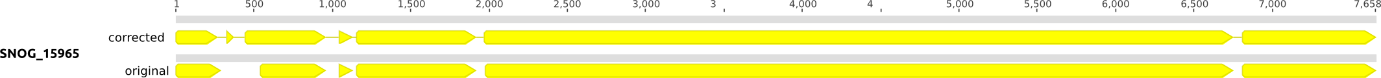
*
