# Supplementary material for: Comprehensive Annotation of the Parastagonospora nodorum Reference Genome Using Next-Generation Genomics, Transcriptomics and Proteogenomics
Source: PLoS One. 2016 Feb 3;11(2):e0147221. doi: 10.1371/journal.pone.0147221 (PMC4739733; doi:10.1371/journal.pone.0147221)
Supplement: S1 Table — (DOCX) [file pone.0147221.s002.docx]

## S1 Table | Repeat Content

The repeat content did not substantially differ between genome assemblies

| Repeat class | Old count | New count | Delta | Delta% |
| --- | --- | --- | --- | --- |
| Subtelomeric |  |  |  |  |
| R22 | 12252 | 12645 | 393 | 3.20764 |
| X15 | 87189 | 87249 | 60 | 0.068816 |
| X26 | 76622 | 77179 | 557 | 0.726945 |
| X35 | 14136 | 14135 | -1 | -0.00707414 |
| X48 | 5377 | 5593 | 216 | 4.01711 |
| Ribosomal |  |  |  |  |
| Y1 | 400707 | 400875 | 168 | 0.0419259 |
| Other |  |  |  |  |
| ELSA | 34285 | 34319 | 34 | 0.0991687 |
| MOLLY | 49213 | 49296 | 83 | 0.168655 |
| PIXIE | 38612 | 38635 | 23 | 0.059567 |
| R10 | 43944 | 43835 | -109 | -0.248043 |
| R25 | 43858 | 44107 | 249 | 0.567741 |
| R31 | 39053 | 39429 | 376 | 0.962794 |
| R37 | 106258 | 106392 | 134 | 0.126108 |
| R38 | 8760 | 8827 | 67 | 0.76484 |
| R39 | 36613 | 36778 | 165 | 0.45066 |
| R51 | 25640 | 25863 | 223 | 0.869735 |
| R8 | 277650 | 277645 | -5 | -0.00180083 |
| R9 | 163980 | 162676 | -1304 | -0.795219 |
| X0 | 149537 | 147850 | -1687 | -1.12815 |
| X11 | 126539 | 126536 | -3 | -0.00237081 |
| X12 | 24813 | 24909 | 96 | 0.386894 |
| X23 | 12354 | 12348 | -6 | -0.0485673 |
| X28 | 28414 | 28248 | -166 | -0.584219 |
| X3 | 464053 | 463844 | -209 | -0.045038 |
| X36 | 5067 | 5107 | 40 | 0.789422 |
| X46 | 1315 | 1315 | 0 | 0 |
| X96 | 4320 | 4311 | -9 | -0.208333 |
| *Sum* | 2280561 | 2279946 | -615 | 10.239207 |
