# Supplementary material for: Comprehensive Annotation of the Parastagonospora nodorum Reference Genome Using Next-Generation Genomics, Transcriptomics and Proteogenomics
Source: PLoS One. 2016 Feb 3;11(2):e0147221. doi: 10.1371/journal.pone.0147221 (PMC4739733; doi:10.1371/journal.pone.0147221)
Supplement: S2 Table — A) Genes that are a product of merging two or more annotations. B) Genes that are a product of splitting one annotation into two or more genes. (DOCX) [file pone.0147221.s003.docx]

## S2a | List of genes that are a product of merging two or more genes

| SNOG_30955 | SNOG_30946 | SNOG_30935 | SNOG_30934 |
| --- | --- | --- | --- |
| SNOG_30930 | SNOG_30889 | SNOG_30841 | SNOG_30798 |
| SNOG_30795 | SNOG_30785 | SNOG_30783 | SNOG_30773 |
| SNOG_30761 | SNOG_30760 | SNOG_30747 | SNOG_30727 |
| SNOG_30715 | SNOG_30704 | SNOG_30693 | SNOG_30688 |
| SNOG_30682 | SNOG_30649 | SNOG_30643 | SNOG_30629 |
| SNOG_30627 | SNOG_30611 | SNOG_30605 | SNOG_30602 |
| SNOG_30583 | SNOG_30581 | SNOG_30578 | SNOG_30574 |
| SNOG_30573 | SNOG_30568 | SNOG_30557 | SNOG_30556 |
| SNOG_30549 | SNOG_30546 | SNOG_30537 | SNOG_30536 |
| SNOG_30534 | SNOG_30488 | SNOG_30483 | SNOG_30473 |
| SNOG_30472 | SNOG_30449 | SNOG_30372 | SNOG_30366 |
| SNOG_30357 | SNOG_30342 | SNOG_30324 | SNOG_30271 |
| SNOG_30247 | SNOG_30094 | SNOG_30040 |  |

## S2b | List of genes that are a product of splitting one annotation into two

| SNOG_30991 | SNOG_30987 | SNOG_30986 | SNOG_30984 |
| --- | --- | --- | --- |
| SNOG_30983 | SNOG_30982 | SNOG_30981 | SNOG_30980 |
| SNOG_30979 | SNOG_30978 | SNOG_30977 | SNOG_30975 |
| SNOG_30965 | SNOG_30963 | SNOG_30961 | SNOG_30954 |
| SNOG_30953 | SNOG_30951 | SNOG_30950 | SNOG_30949 |
| SNOG_30947 | SNOG_30942 | SNOG_30938 | SNOG_30933 |
| SNOG_30931 | SNOG_30929 | SNOG_30928 | SNOG_30927 |
| SNOG_30926 | SNOG_30924 | SNOG_30922 | SNOG_30918 |
| SNOG_30917 | SNOG_30916 | SNOG_30915 | SNOG_30914 |
| SNOG_30913 | SNOG_30912 | SNOG_30911 | SNOG_30910 |
| SNOG_30909 | SNOG_30904 | SNOG_30898 | SNOG_30897 |
| SNOG_30896 | SNOG_30895 | SNOG_30894 | SNOG_30892 |
| SNOG_30891 | SNOG_30890 | SNOG_30886 | SNOG_30883 |
| SNOG_30882 | SNOG_30879 | SNOG_30877 | SNOG_30876 |
| SNOG_30875 | SNOG_30874 | SNOG_30873 | SNOG_30872 |
| SNOG_30871 | SNOG_30870 | SNOG_30868 | SNOG_30867 |
| SNOG_30866 | SNOG_30865 | SNOG_30860 | SNOG_30859 |
| SNOG_30857 | SNOG_30856 | SNOG_30855 | SNOG_30853 |
| SNOG_30847 | SNOG_30844 | SNOG_30843 | SNOG_30840 |
| SNOG_30832 | SNOG_30830 | SNOG_30829 | SNOG_30827 |
| SNOG_30826 | SNOG_30811 | SNOG_30809 | SNOG_30808 |
| SNOG_30805 | SNOG_30804 | SNOG_30800 | SNOG_30799 |
| SNOG_30797 | SNOG_30796 | SNOG_30792 | SNOG_30787 |
| SNOG_30786 | SNOG_30782 | SNOG_30776 | SNOG_30770 |
| SNOG_30774 | SNOG_30771 | SNOG_30768 | SNOG_30767 |
| SNOG_30757 | SNOG_30756 | SNOG_30755 | SNOG_30754 |
| SNOG_30753 | SNOG_30752 | SNOG_30750 | SNOG_30749 |
| SNOG_30746 | SNOG_30745 | SNOG_30744 | SNOG_30743 |
| SNOG_30742 | SNOG_30740 | SNOG_30738 | SNOG_30736 |
| SNOG_30735 | SNOG_30733 | SNOG_30732 | SNOG_30730 |
| SNOG_30729 | SNOG_30724 | SNOG_30720 | SNOG_30718 |
| SNOG_30714 | SNOG_30713 | SNOG_30712 | SNOG_30711 |
| SNOG_30709 | SNOG_30700 | SNOG_30694 | SNOG_30690 |
| SNOG_30689 | SNOG_30687 | SNOG_30681 | SNOG_30676 |
| SNOG_30665 | SNOG_30663 | SNOG_30656 | SNOG_30653 |
| SNOG_30652 | SNOG_30650 | SNOG_30642 | SNOG_30640 |
| SNOG_30636 | SNOG_30634 | SNOG_30626 | SNOG_30624 |
| SNOG_30623 | SNOG_30622 | SNOG_30620 | SNOG_30618 |
| SNOG_30617 | SNOG_30616 | SNOG_30613 | SNOG_30606 |
| SNOG_30604 | SNOG_30600 | SNOG_30593 | SNOG_30591 |
| SNOG_30590 | SNOG_30587 | SNOG_30579 | SNOG_30576 |
| SNOG_30575 | SNOG_30572 | SNOG_30567 | SNOG_30562 |
| SNOG_30555 | SNOG_30554 | SNOG_30552 | SNOG_30548 |
| SNOG_30547 | SNOG_30541 | SNOG_30540 | SNOG_30539 |
| SNOG_30527 | SNOG_30520 | SNOG_30519 | SNOG_30514 |
| SNOG_30513 | SNOG_30512 | SNOG_30511 | SNOG_30505 |
| SNOG_30500 | SNOG_30496 | SNOG_30495 | SNOG_30492 |
| SNOG_30491 | SNOG_30475 | SNOG_30471 | SNOG_30469 |
| SNOG_30462 | SNOG_30460 | SNOG_30457 | SNOG_30456 |
| SNOG_30455 | SNOG_30454 | SNOG_30448 | SNOG_30447 |
| SNOG_30446 | SNOG_30441 | SNOG_30438 | SNOG_30431 |
| SNOG_30430 | SNOG_30428 | SNOG_30426 | SNOG_30425 |
| SNOG_30422 | SNOG_30421 | SNOG_30417 | SNOG_30416 |
| SNOG_30412 | SNOG_30409 | SNOG_30402 | SNOG_30401 |
| SNOG_30400 | SNOG_30394 | SNOG_30393 | SNOG_30392 |
| SNOG_30391 | SNOG_30389 | SNOG_30387 | SNOG_30377 |
| SNOG_30376 | SNOG_30373 | SNOG_30370 | SNOG_30368 |
| SNOG_30356 | SNOG_30353 | SNOG_30351 | SNOG_30336 |
| SNOG_30333 | SNOG_30330 | SNOG_30319 | SNOG_30315 |
| SNOG_30313 | SNOG_30312 | SNOG_30302 | SNOG_30299 |
| SNOG_30295 | SNOG_30292 | SNOG_30291 | SNOG_30290 |
| SNOG_30286 | SNOG_30282 | SNOG_30280 | SNOG_30279 |
| SNOG_30277 | SNOG_30276 | SNOG_30275 | SNOG_30274 |
| SNOG_30272 | SNOG_30267 | SNOG_30263 | SNOG_30261 |
| SNOG_30260 | SNOG_30257 | SNOG_30256 | SNOG_30252 |
| SNOG_30246 | SNOG_30245 | SNOG_30242 | SNOG_30241 |
| SNOG_30240 | SNOG_30239 | SNOG_30238 | SNOG_30237 |
| SNOG_30236 | SNOG_30231 | SNOG_30230 | SNOG_30223 |
| SNOG_30218 | SNOG_30217 | SNOG_30213 | SNOG_30212 |
| SNOG_30211 | SNOG_30206 | SNOG_30202 | SNOG_30192 |
| SNOG_30188 | SNOG_30185 | SNOG_30179 | SNOG_30175 |
| SNOG_30992 | SNOG_30171 | SNOG_30170 | SNOG_30169 |
| SNOG_30167 | SNOG_30164 | SNOG_30158 | SNOG_30155 |
| SNOG_30154 | SNOG_30153 | SNOG_30152 | SNOG_30151 |
| SNOG_30149 | SNOG_30147 | SNOG_30143 | SNOG_30139 |
| SNOG_30138 | SNOG_30131 | SNOG_30130 | SNOG_30129 |
| SNOG_30126 | SNOG_30125 | SNOG_30122 | SNOG_30118 |
| SNOG_30116 | SNOG_30113 | SNOG_30110 | SNOG_30109 |
| SNOG_30108 | SNOG_30107 | SNOG_30106 | SNOG_30105 |
| SNOG_30103 | SNOG_30097 | SNOG_30091 | SNOG_30090 |
| SNOG_30089 | SNOG_30087 | SNOG_30086 | SNOG_30084 |
| SNOG_30082 | SNOG_30081 | SNOG_30080 | SNOG_30075 |
| SNOG_30071 | SNOG_30069 | SNOG_30068 | SNOG_30066 |
| SNOG_30062 | SNOG_30061 | SNOG_30060 | SNOG_30059 |
| SNOG_30058 | SNOG_30057 | SNOG_30056 | SNOG_30053 |
| SNOG_30052 | SNOG_30050 | SNOG_30049 | SNOG_30047 |
| SNOG_30045 | SNOG_30043 | SNOG_30042 | SNOG_30041 |
| SNOG_30032 | SNOG_30028 | SNOG_30027 | SNOG_30020 |
| SNOG_30017 | SNOG_30016 | SNOG_30012 | SNOG_30011 |
