# Supplementary material for: Comprehensive Annotation of the Parastagonospora nodorum Reference Genome Using Next-Generation Genomics, Transcriptomics and Proteogenomics
Source: PLoS One. 2016 Feb 3;11(2):e0147221. doi: 10.1371/journal.pone.0147221 (PMC4739733; doi:10.1371/journal.pone.0147221)
Supplement: S3 Table — (DOCX) [file pone.0147221.s004.docx]

## S3 | Genes at new loci

| SNOG_30993 | SNOG_30990 | SNOG_30989 | SNOG_30988 |
| --- | --- | --- | --- |
| SNOG_30976 | SNOG_30974 | SNOG_30973 | SNOG_30972 |
| SNOG_30971 | SNOG_30968 | SNOG_30967 | SNOG_30966 |
| SNOG_30964 | SNOG_30962 | SNOG_30960 | SNOG_30959 |
| SNOG_30957 | SNOG_30956 | SNOG_30952 | SNOG_30948 |
| SNOG_30945 | SNOG_30944 | SNOG_30941 | SNOG_30940 |
| SNOG_30937 | SNOG_30936 | SNOG_30932 | SNOG_30923 |
| SNOG_30921 | SNOG_30920 | SNOG_30919 | SNOG_30908 |
| SNOG_30907 | SNOG_30906 | SNOG_30905 | SNOG_30903 |
| SNOG_30902 | SNOG_30901 | SNOG_30900 | SNOG_30899 |
| SNOG_30994 | SNOG_30893 | SNOG_30888 | SNOG_30887 |
| SNOG_30884 | SNOG_30880 | SNOG_30878 | SNOG_30869 |
| SNOG_30864 | SNOG_30863 | SNOG_30862 | SNOG_30861 |
| SNOG_30858 | SNOG_30854 | SNOG_30852 | SNOG_30851 |
| SNOG_30850 | SNOG_30848 | SNOG_30846 | SNOG_30845 |
| SNOG_30842 | SNOG_30838 | SNOG_30837 | SNOG_30836 |
| SNOG_30835 | SNOG_30834 | SNOG_30833 | SNOG_30831 |
| SNOG_30828 | SNOG_30825 | SNOG_30824 | SNOG_30822 |
| SNOG_30821 | SNOG_30819 | SNOG_30818 | SNOG_30810 |
| SNOG_30807 | SNOG_30806 | SNOG_30803 | SNOG_30802 |
| SNOG_30801 | SNOG_30794 | SNOG_30793 | SNOG_30791 |
| SNOG_30790 | SNOG_30788 | SNOG_30784 | SNOG_30781 |
| SNOG_30780 | SNOG_30779 | SNOG_30778 | SNOG_30777 |
| SNOG_30772 | SNOG_30769 | SNOG_30766 | SNOG_30765 |
| SNOG_30764 | SNOG_30763 | SNOG_30762 | SNOG_30759 |
| SNOG_30758 | SNOG_30751 | SNOG_30748 | SNOG_30741 |
| SNOG_30739 | SNOG_30731 | SNOG_30728 | SNOG_30725 |
| SNOG_30723 | SNOG_30722 | SNOG_30721 | SNOG_30719 |
| SNOG_30717 | SNOG_30716 | SNOG_30710 | SNOG_30708 |
| SNOG_30707 | SNOG_30706 | SNOG_30705 | SNOG_30703 |
| SNOG_30701 | SNOG_30699 | SNOG_30698 | SNOG_30697 |
| SNOG_30696 | SNOG_30695 | SNOG_30692 | SNOG_30691 |
| SNOG_30686 | SNOG_30684 | SNOG_30679 | SNOG_30678 |
| SNOG_30677 | SNOG_30675 | SNOG_30674 | SNOG_30673 |
| SNOG_30672 | SNOG_30671 | SNOG_30670 | SNOG_30669 |
| SNOG_30668 | SNOG_30667 | SNOG_30666 | SNOG_30664 |
| SNOG_30662 | SNOG_30661 | SNOG_30659 | SNOG_30658 |
| SNOG_30657 | SNOG_30654 | SNOG_30648 | SNOG_30647 |
| SNOG_30646 | SNOG_30645 | SNOG_30637 | SNOG_30633 |
| SNOG_30632 | SNOG_30631 | SNOG_30630 | SNOG_30625 |
| SNOG_30621 | SNOG_30619 | SNOG_30615 | SNOG_30614 |
| SNOG_30612 | SNOG_30610 | SNOG_30609 | SNOG_30608 |
| SNOG_30607 | SNOG_30603 | SNOG_30601 | SNOG_30599 |
| SNOG_30597 | SNOG_30596 | SNOG_30595 | SNOG_30594 |
| SNOG_30592 | SNOG_30589 | SNOG_30588 | SNOG_30585 |
| SNOG_30584 | SNOG_30582 | SNOG_30577 | SNOG_30571 |
| SNOG_30569 | SNOG_30564 | SNOG_30563 | SNOG_30560 |
| SNOG_30559 | SNOG_30558 | SNOG_30553 | SNOG_30550 |
| SNOG_30545 | SNOG_30544 | SNOG_30543 | SNOG_30542 |
| SNOG_30532 | SNOG_30530 | SNOG_30529 | SNOG_30528 |
| SNOG_30526 | SNOG_30525 | SNOG_30524 | SNOG_30523 |
| SNOG_30522 | SNOG_30518 | SNOG_30516 | SNOG_30515 |
| SNOG_30510 | SNOG_30509 | SNOG_30508 | SNOG_30507 |
| SNOG_30504 | SNOG_30503 | SNOG_30502 | SNOG_30501 |
| SNOG_30499 | SNOG_30498 | SNOG_30497 | SNOG_30494 |
| SNOG_30493 | SNOG_30490 | SNOG_30489 | SNOG_30487 |
| SNOG_30486 | SNOG_30485 | SNOG_30484 | SNOG_30482 |
| SNOG_30481 | SNOG_30480 | SNOG_30479 | SNOG_30478 |
| SNOG_30477 | SNOG_30476 | SNOG_30474 | SNOG_30468 |
| SNOG_30467 | SNOG_30466 | SNOG_30465 | SNOG_30464 |
| SNOG_30463 | SNOG_30461 | SNOG_30459 | SNOG_30453 |
| SNOG_30452 | SNOG_30451 | SNOG_30450 | SNOG_30445 |
| SNOG_30442 | SNOG_30440 | SNOG_30439 | SNOG_30436 |
| SNOG_30434 | SNOG_30433 | SNOG_30432 | SNOG_30429 |
| SNOG_30424 | SNOG_30423 | SNOG_30420 | SNOG_30419 |
| SNOG_30418 | SNOG_30415 | SNOG_30413 | SNOG_30410 |
| SNOG_30407 | SNOG_30406 | SNOG_30405 | SNOG_30403 |
| SNOG_30399 | SNOG_30398 | SNOG_30396 | SNOG_30395 |
| SNOG_30390 | SNOG_30388 | SNOG_30386 | SNOG_30385 |
| SNOG_30384 | SNOG_30383 | SNOG_30382 | SNOG_30380 |
| SNOG_30379 | SNOG_30378 | SNOG_30374 | SNOG_30369 |
| SNOG_30367 | SNOG_30365 | SNOG_30364 | SNOG_30363 |
| SNOG_30362 | SNOG_30361 | SNOG_30360 | SNOG_30359 |
| SNOG_30358 | SNOG_30355 | SNOG_30354 | SNOG_30352 |
| SNOG_30350 | SNOG_30349 | SNOG_30348 | SNOG_30347 |
| SNOG_30346 | SNOG_30344 | SNOG_30343 | SNOG_30341 |
| SNOG_30340 | SNOG_30339 | SNOG_30337 | SNOG_30335 |
| SNOG_30334 | SNOG_30332 | SNOG_30331 | SNOG_30329 |
| SNOG_30328 | SNOG_30327 | SNOG_30326 | SNOG_30325 |
| SNOG_30323 | SNOG_30322 | SNOG_30321 | SNOG_30320 |
| SNOG_30318 | SNOG_30317 | SNOG_30316 | SNOG_30314 |
| SNOG_30311 | SNOG_30310 | SNOG_30309 | SNOG_30308 |
| SNOG_30307 | SNOG_30306 | SNOG_30305 | SNOG_30304 |
| SNOG_30303 | SNOG_30300 | SNOG_30297 | SNOG_30294 |
| SNOG_30289 | SNOG_30288 | SNOG_30287 | SNOG_30285 |
| SNOG_30284 | SNOG_30283 | SNOG_30281 | SNOG_30278 |
| SNOG_30273 | SNOG_30270 | SNOG_30269 | SNOG_30268 |
| SNOG_30266 | SNOG_30265 | SNOG_30264 | SNOG_30258 |
| SNOG_30255 | SNOG_30253 | SNOG_30250 | SNOG_30249 |
| SNOG_30248 | SNOG_30244 | SNOG_30235 | SNOG_30234 |
| SNOG_30233 | SNOG_30232 | SNOG_30229 | SNOG_30228 |
| SNOG_30227 | SNOG_30225 | SNOG_30224 | SNOG_30221 |
| SNOG_30220 | SNOG_30219 | SNOG_30216 | SNOG_30215 |
| SNOG_30214 | SNOG_30210 | SNOG_30209 | SNOG_30208 |
| SNOG_30207 | SNOG_30205 | SNOG_30204 | SNOG_30203 |
| SNOG_30201 | SNOG_30200 | SNOG_30199 | SNOG_30198 |
| SNOG_30197 | SNOG_30196 | SNOG_30195 | SNOG_30194 |
| SNOG_30193 | SNOG_30190 | SNOG_30186 | SNOG_30182 |
| SNOG_30180 | SNOG_30178 | SNOG_30177 | SNOG_30176 |
| SNOG_30174 | SNOG_30157 | SNOG_30148 | SNOG_30145 |
| SNOG_30137 | SNOG_30134 | SNOG_30132 | SNOG_30128 |
| SNOG_30124 | SNOG_30123 | SNOG_30121 | SNOG_30120 |
| SNOG_30119 | SNOG_30115 | SNOG_30114 | SNOG_30112 |
| SNOG_30111 | SNOG_30104 | SNOG_30102 | SNOG_30101 |
| SNOG_30100 | SNOG_30099 | SNOG_30098 | SNOG_30096 |
| SNOG_30095 | SNOG_30093 | SNOG_30092 | SNOG_30085 |
| SNOG_30079 | SNOG_30078 | SNOG_30077 | SNOG_30074 |
| SNOG_30073 | SNOG_30072 | SNOG_30070 | SNOG_30067 |
| SNOG_30065 | SNOG_30064 | SNOG_30063 | SNOG_30054 |
| SNOG_30048 | SNOG_30046 | SNOG_30044 | SNOG_30039 |
| SNOG_30036 | SNOG_30035 | SNOG_30034 | SNOG_30033 |
| SNOG_30031 | SNOG_30030 | SNOG_30029 | SNOG_30026 |
| SNOG_30025 | SNOG_30024 | SNOG_30022 | SNOG_30019 |
| SNOG_30014 | SNOG_30013 | SNOG_30008 |  |
