# Supplementary material for: Comprehensive Annotation of the Parastagonospora nodorum Reference Genome Using Next-Generation Genomics, Transcriptomics and Proteogenomics
Source: PLoS One. 2016 Feb 3;11(2):e0147221. doi: 10.1371/journal.pone.0147221 (PMC4739733; doi:10.1371/journal.pone.0147221)
Supplement: S4 Table — (DOCX) [file pone.0147221.s005.docx]

## S4a Table | Details of cysteine-rich protein properties

| Gene name | Protein length | Cysteine count | Cysteine percentage | BLAST matches |
| --- | --- | --- | --- | --- |
| SNOG_30077 | 66 | 9 | 13.6 | No |
| SNOG_30525 | 74 | 10 | 13.5 | No |
| SNOG_30316 | 94 | 11 | 11.7 | No |
| SNOG_30335 | 70 | 8 | 11.4 | No |
| SNOG_30888 | 53 | 6 | 11.3 | No |
| SNOG_30837 | 56 | 6 | 10.7 | No |
| SNOG_30741 | 355 | 37 | 10.4 | Carbohydrate-binding |
| SNOG_30253 | 58 | 6 | 10.3 | No |
| SNOG_30352 | 79 | 8 | 10.1 | No |
| SNOG_30019 | 60 | 6 | 10 | No |
| SNOG_30451 | 62 | 6 | 9.7 | Fungal hypothetical genes |
| SNOG_30925 | 104 | 10 | 9.6 | No |
| SNOG_30828 | 84 | 8 | 9.5 | No |
| SNOG_30466 | 84 | 8 | 9.5 | Tox1 |
| SNOG_30530 | 76 | 7 | 9.2 | No |
| SNOG_30989 | 55 | 5 | 9.1 | No |

## S4b Table | Matches to PHIbase

| PHIbase Class | New proteins | Modified proteins |
| --- | --- | --- |
| Mutants are lethal | 2 | 3 |
| Mutants have reduced virulence | 20 | 120 |
| Mutants show mixed results | 1 | 17 |
| Effector (plant avirulence determinant) | 0 | 2 |
| Mutants have lost pathogenicity | 3 | 11 |
| Mutants have increased virulence | 0 | 4 |
| Chemistry target (unknown phenotype) | 0 | 4 |

## S4c Table | Changes in detected conserved protein domains

| Domain ID | Domain name | Original Protein Count | Corrected Protein Count |
| --- | --- | --- | --- |
| Putative pathogenicity-related domains | | | |
| PF14856 | HCE2 - Pathogen effector; putative necrosis-inducing factor | 1 | 2 |
| PF00652 | Ricin-type beta-trefoil lectin | 0 | 1 |
| Top 5 domains with increased hits | | | |
| IPR001138 | Zn(2)-C6 fungal-type DNA-binding domain | 89 | 206 |
| IPR007219 | Transcription factor domain | 91 | 142 |
| IPR000719 | Protein kinase domain | 120 | 157 |
| IPR001810 | F-box domain | 49 | 77 |
| IPR001002 | Chitin-binding | 14 | 35 |
