# Supplementary material for: Comprehensive Annotation of the Parastagonospora nodorum Reference Genome Using Next-Generation Genomics, Transcriptomics and Proteogenomics
Source: PLoS One. 2016 Feb 3;11(2):e0147221. doi: 10.1371/journal.pone.0147221 (PMC4739733; doi:10.1371/journal.pone.0147221)
Supplement: S1 Text — (DOCX) [file pone.0147221.s006.docx]

## S1 Text | Cutadapt Parameters

Cutadapt was run trimming bases below a quality cutoff of Phred Score 25, using known Illumina adapters and discarding trimmed reads where the final length was less than 50 bp.

set -o errexit

set -o nounset

set -o xtrace

set -o pipefail

read1_base=`basename ${1}`

read2_base=`basename ${2}`

prefix=`printf "%s\n%s\n" "$read1_base" "${read2_base}" | sed -e 'N;s/^\(.*\).*\n\1.*$/\1/'`

strainID=${prefix::-1}

echo "Running cutadapt (first pass) - $strainID"

cutadapt \

--quality-cutoff=25 \

--adapter=CTGTCTCTTATACACATCTCCGAGCCCACGAGAC \

--minimum-length 50 \

-o tmp.${strainID}.1.fastq \

-p tmp.${strainID}.2.fastq \

$1 \

$2 \

> ${strainID}.report_1.txt

echo "Running cutadapt (second pass) - $strainID"

cutadapt \

--quality-cutoff=25 \

--adapter=CTGTCTCTTATACACATCTGACGCTGCCGACGA \

--minimum-length 50 \

-o ${strainID}.2.trimmed.fastq \

-p ${strainID}.1.trimmed.fastq \

tmp.${strainID}.2.fastq tmp.${strainID}.1.fastq \

> ${strainID}.report_2.txt

rm tmp.${strainID}.1.fastq tmp.${strainID}.2.fastq

gzip ${strainID}*.trimmed.fastq

tar -czvf ${strainID}.cutadapt.reports.tgz ${strainID}.report*.txt

rm ${strainID}.report*.txt
